# Pediatric Spina Bifida and Spinal Cord Injury

**DOI:** 10.3390/jpm12060985

**Published:** 2022-06-17

**Authors:** Joslyn Gober, Sruthi P. Thomas, David R. Gater

**Affiliations:** 1 Department of Physical Medicine and Rehabilitation, Miller School of Medicine, University of Miami, Miami, FL 33136, USA; dgater@miami.edu; 2 The Christine E. Lynn Rehabilitation Center for the Miami Project to Cure Paralysis, Miami, FL 33136, USA; 3 Departments of Physical Medicine & Rehabilitation and Neurosurgery, Baylor College of Medicine, Houston, TX 77030, USA; spthomas@texaschildrens.org; 4 The Miami Project to Cure Paralysis, Miller School of Medicine, University of Miami, Miami, FL 33136, USA

**Keywords:** spina bifida, spinal cord injury, pediatrics, neurogenic bladder, neurogenic bowel, autonomic dysreflexia

## Abstract

Pediatric spina bifida (SB) and spinal cord injury (SCI) are unfortunately common in our society, and their unique findings and comorbidities warrant special consideration. This manuscript will discuss the epidemiology, pathophysiology, prevention, and management strategies for children growing and developing with these unique neuromuscular disorders. Growth and development of the maturing child places them at high risk of spinal cord tethering, syringomyelia, ascending paralysis, pressure injuries, and orthopedic abnormalities that must be addressed frequently and judiciously. Similarly, proper neurogenic bladder and neurogenic bowel management is essential not just for medical safety, but also for optimal psychosocial integration into the child’s expanding social circle.

## 1. Spina Bifida

### 1.1. Introduction

Spina bifida (SB) is one of the most common childhood disabilities [1]. It is a birth defect that affects the spine due to a neural tube defect (NTD) with incomplete closure and can occur anywhere along the spine if the neural tube does not close all the way. When this occurs, the vertebra that protects the spinal cord does not form and close properly, resulting in damage to the spinal cord and nerves [2]. It has been associated with increased mortality and disability and may require lifelong medical care [3]. Each year, approximately 1427 babies are born with SB in the United States, or 1 in every 2758 live births [4], although the incidence has decreased over the last several decades. This reduction can be attributed to folic acid supplementation and early detection [2]. SB may range from mild to severe, with no resulting disabilities to significant impairments, depending on the extent and location of the defect.

### 1.2. Pathophysiology & Epidemiology

NTDs develop as a consequence of abnormal embryologic development [5]. They are classified based on the degree and pattern of neuroectodermal malformation during embryonic development [6]. During pregnancy, the human brain and spine begin as a flat plate of cells, which roll into the neural tube. Neural tube closure begins at approximately 18 days post-fertilization and completely closes within the first 26–28 days of pregnancy, often before women even know they are pregnant [7]. If all or portions of the neural tube fail to develop or close properly, an opening remains, resulting in a neural tube defect. The rostral portion of the neural tube develops to form the brain and spinal cord. Defects that occur during this time frame result in open NTD.

There are multifactorial risks for NTDs, including nutrition, environment, and genetics. Nutritionally, folic acid is a proven intervention to prevent NTD, although the exact mechanism by which it does so is unclear [8]. Nonetheless, due to consistent clinical evidence, the U.S. Centers for Disease Control and Prevention (CDC) recommend that all women of childbearing age consume 400 mcg of folic acid per day, and women with a history of SB or a previous pregnancy affected by NTD should take 4000 mcg of folic acid daily [2]. Environmental risk factors include certain medications that have been shown to alter folate metabolism, such as valproate, methotrexate, and rifampin, as well as maternal medical comorbidities such as diabetes mellitus, obesity, cigarette smoking, and hyperthermia [6]. Genetics play a role as well with higher rates of SB found with consanguinity; individuals with previously affected pregnancies are three to five times more likely to have recurrence in a subsequent pregnancy [9]. There have been racial and ethnic variabilities noted, with Hispanics demonstrating higher prevalence when compared to non-Hispanic white mothers [10]. This has been attributed to differences in folic acid consumption, genetic factors affecting folic acid metabolism, and lower rates of elective termination [11]. Additionally, there is thought to be gene variants that are more susceptible to the adverse effects the environment (pollution) which may contribute to an increased risk of abnormal embryologic development [12].

It is estimated that 220,000 neonates are born with neural tube defects worldwide annually, with a large percentage in developing countries due to malnutrition [9]. In the United States, the prevalence of SB and other neural tube defects have decreased significantly following the mandatory folic acid fortification of certain food products as well as early detection strategies [13].

### 1.3. Types of Spina Bifida

There are three common types of SB based upon the degree of neural tube closure.

#### 1.3.1. Spina Bifida Occulta

Spina Bifida occulta is the mildest type in which there is no NTD but rather an abnormal formation of posterior vertebra. In SB occulta, the spinal cord and nerves are unaffected, resulting in no significant disability. In fact, this type of SB often goes unnoticed, or may be diagnosed incidentally. Sometimes there may be a tuft of hair, a small dimple, or a vascular nevus in the region of the abnormality [14].

#### 1.3.2. Meningocele

Meningocele is the moderate form of SB in which a sack of fluid protrudes through an opening in the neural tube and defect in the vertebral column. In these cases, the sac contains cerebrospinal fluid but does not contain spinal cord or nerves, such that there is typically little or no neurologic damage, and unlikely disability.

#### 1.3.3. Myelomeningocele

The most severe and unfortunately the most common type of SB is the myelomeningocele that is among the most complex congenital anomalies compatible with life [15]. With this entity, not only is there a sack of fluid protruding through the opening of the vertebral defect, but the sack contains portions of spinal cord and nerves. Myelomeningocele subsequently shows varying degrees of neurologic deficit depending on the level and extent of the defect [16]. For the purpose of the remainder of this chapter, SB will be used when referring to myelomeningocele.

### 1.4. Early Diagnosis

SB may be diagnosed during pregnancy or soon after the baby is born. With advancements in technology and diagnostic tools, SB is commonly found during prenatal screening visits. Alpha-fetoprotein (AFP) is the most prominent protein in a fetus, and it can be measured in maternal blood. While not pathognomonic for SB, a high AFP level may be indicative of an open neural tube defect leaking extra AFP into maternal serum; additional testing is warranted [17]. Physicians can perform an amniocentesis to directly evaluate the levels of AFP in the amniotic fluid. More recently, ultrasound (US) has been demonstrated to be the most accurate and common method utilized to detect SB [18]. With high resolution US, it is possible to determine the defect’s spinal level by localizing vertebral arch defects, while also evaluating for concomitant conditions. The spinal US view reveals the spinal defect and can be seen both on both longitudinal and transverse views. The cranial US views may reveal frontal bossing, known as the “lemon sign,” or compressed cerebellar hemispheres, known as the “banana sign,” representing hydrocephalus and Chiari II malformations, respectively [19]. These anomalies may be detected by US as early as the late first trimester. Finally, fetal magnetic resonance imaging (MRI) can provide high-resolution visualization of the structures when more detail is required [19].

In some cases, SB may go undiagnosed until after the baby is born due to poor prenatal care or subtle findings on imaging that had gone unnoticed. In these cases, there may be a hairy patch of skin or a dimple on the newborn’s back, warranting additional postnatal imaging to provide a better picture.

### 1.5. NTD Closure/Repair

Early SB identification during pregnancy allows for multiple management options, beginning with the decision of whether or not to continue with the pregnancy, appropriate prenatal care, potential for surgical interventions, and delivery planning. Lesions may be closed surgically at the prenatal stage or immediately at the postnatal stage. When repaired in-utero, a minimally invasive approach via open uterine incision or fetoscopic surgery can be performed. Conversely, when performed postnatally, the lesions are closed within 48 h after birth, and the infant should be evaluated frequently for signs of hydrocephalus [20]. There are risks and benefits to each of the interventions. The Management of Meningocele Study (MOMS) in 2011 was a prospective, randomized controlled trial that provided level 1 evidence comparing the two interventions. Risks associated with prenatal closure included high rates of premature labor, infections, and maternal complications such as uterine dehiscence in the setting of open uterine incisions; benefits of prenatal closure included improved outcomes in multiple domains [21,22]. The study reported reduced need for shunting with prenatal repair, a reduction in radiographic and symptomatic Chiari II malformations, and improved lower extremity function. It also demonstrated improved composite scores of motor and mental function with better orthopedic outcomes, but no differences in cognitive function [23]. No difference was noted regarding subsequent development of neurogenic bladder with need for intermittent catheterization [22]. The 30-month longitudinal follow up showed sustained improvements, but there appeared to be an increase in tethered cord following prenatal closure, without noted offsets of motor function [22]. A systemic review of neurodevelopmental outcomes demonstrated similar risks of neurodevelopment impairments in infants between the two groups, despite an increased risk of prematurity noted in the prenatal compared to the postnatal repair [23].

### 1.6. Presentation 

Children with SB have a range of phenotypes, dependent on location and extent of lesion. It is important to identify the functional motor level, not just the anatomical level, as they are not always the same. The functional motor level allows the medical and therapeutic team to anticipate management and help guide the patient and family to monitor for potential complications, provide orthotic prescriptions, and predict equipment needs for optimizing independence [24]. Children with SB will experience delays in their early gross motor milestones, and it is generally believed that children will achieve their maximum ambulatory potential by 9 years of age [25]. As children with SB enter adolescence, they experience substantial growth and weight gains, leading to increased physical demands and inability to keep up with their colleagues. As a result, there is often an increased reliance on wheelchair devices for mobility as they progress through adolescence and adulthood [24,26,27].

The level of the SB lesion roughly correlates to the spinal cord levels, with most having flaccid lower extremity paralysis below the level of lesion, although sacral elements may be hyperreflexic depending on the neurosurgical repair [28,29]. The higher the level of involvement (cervical lesions) result in more significant impairments as well as increased risks for those complications commonly seen in SB, including hydrocephalus, neuromuscular scoliosis, and musculoskeletal contractures. Individuals with thoracic lesions usually gain full use of their upper extremities, however, often demonstrate respiratory insufficiency due to partial innervation of abdominal and intercostal muscles. Children with either cervical and thoracic SB will have a weak trunk, neuromuscular kyphoscoliosis, and complete leg paralysis, with subsequent positional contracture development. They are typically nonfunctional for ambulation but may use a standing frame and are often provided extensive orthotic devices such as trunk-hip-knee-ankle-foot orthoses (THKAFO). Those with high lumber SB, such as L1–L3, usually gain some lower extremity strength, including hip flexion, hip adduction, and possibly partial knee extension. As such, they may be household ambulators with the proper assistive devices, including walkers, forearm (Lofstrand) crutches, reciprocal gait orthoses (RGO), or hip-knee-ankle-foot orthoses (HKAFO). Most, however, typically require wheelchairs for true community mobility. These children are also at risk of early hip dislocation due to muscle imbalance of hip flexors and adductors compared to weak or non-functioning hip extensors and abductors. Mid-lumbar SB, such as L3–L4 lesions, may be household ambulators with a walker, or forearm crutches with knee-ankle-foot orthoses (KAFOs), but are still limited for community ambulation. In addition to hip flexors and adductors, children with low lumbar SB, such as L4–L5 lesions, can also activate knee extensors, ankle dorsiflexors, with some degree of gluteal and hamstring innervation. As a result, these children often have the ability to be both household and community ambulators with forearm crutches and ankle-foot-orthoses (AFO), but likely still require wheelchairs for long distances. Children with sacral SB (S1–S2 lesions) are usually full community ambulators, and may require only supramalleolar or foot orthoses [30]. They often demonstrate pes cavus feet and clawing of toes due to intrinsic muscle denervation; thus, orthotics are used in order to reduce energy expenditure during ambulation. 

### 1.7. Common Comorbidities 

SB causes life-long impairments which require specialty care. A multi-disciplinary clinical setting is the best approach to managing this population. Frequent recurring assessments should be performed with Neurosurgery, Urology, Physical Medicine & Rehabilitation, Orthopedics, Gastroenterology, Nutrition, Endocrinology, and Developmental Pediatricians, as well as therapists, orthotists, nursing, social work, psychology, and eventually transitional specialists to provide care as adults.

#### 1.7.1. Neurosurgery

Historically, at least 80–95% of children with SB develop hydrocephalus [31]. It is one of the most important comorbidities of SB from a neurosurgical perspective, as it can exacerbate other comorbidities such as Chiari II malformation and tethered spinal cord [32]. Symptoms include signs of intracranial pressure, such as headaches, nausea/vomiting, decreased energy, impaired cognition, or high-pitched cries and poor feeding in infants. This symptomatology may overlap those with infections and can be difficult to distinguish at times. Imaging is required and may consist of cranial ultrasounds in infants or brain MRIs. When indicated, neurosurgeons consider placement of a ventriculoperitoneal (VP) shunt or endoscopic third ventriculostomy (ETV); VP shunts are currently the gold standard for the management of hydrocephalus [33]. With the presence of shunts, there is always a risk of complications, including infections and shunt malfunctions. If the child with SB and VPS for hydrocephalus develops symptoms of intracranial pressure, it is generally considered to be a shunt complication until proven otherwise. There is some controversy and practice variability in determining the threshold for these interventions, as some centers will allow larger ventricles. Doing so may reduce shunt implantation and revision rates, and decrease the risk of shunt failure or infection; however, the long-term cognitive outcomes associated with this conservative strategy are unknown [33].

Chiari II malformations are characterized by caudal displacement of the cerebellum and brainstem, causing those elements to herniate into the foramen magnum [33]; these malformations are common in children with SB and hydrocephalus. Chiari II malformations can clinically manifest as a mimic of hydrocephalus but may present with respiratory and/or bulbar compromise and hemiparesis or paraparesis. Depending on the severity of symptoms, the Chiari II malformation may require surgical decompression, but only after shunt issues have been ruled out [34].

In addition to monitoring for hydrocephalus and Chiari II malformations, children should be monitored for signs and symptoms of tethered cords and syringomyelia [35]. Elements of the distal spinal cord may be scarred down (tethered) to the spinal canal, resulting in traction and cord ischemia as the child grows; caudal displacement of the cerebellum and brainstem can also occur in those with Chiari II malformations. Frequent evaluations, particularly during growth spurts, should include evaluation for back pain, increased tone or reflexes, changes in strength or sensation, changes in bowel or bladder function, and evidence of rapid progression of scoliosis. Neurosurgical de-tethering and/or decompression of associated syringomyelia are dependent on the symptoms [36].

#### 1.7.2. Neurourology

A large percentage of individuals with SB will have some degree of neurogenic bladder, such as loss of coordinated micturition as a result of upper motor neuron (UMN) lesions, lower motor neuron (LMN) lesions and/or autonomic dysfunction [29]. The neurogenic bladder may be secondary to spastic pelvic floor, flaccid pelvic floor, overactive detrusor muscle, detrusor sphincter dyssynergia, or a combination of those findings [28]. These features may result in high bladder pressures with a high risk of urinary tract infections, vesicoureteral reflux, hydronephrosis and renal damage. To prevent this, urological and nephrological management should start immediately after birth [28]. Current recommendations include obtaining renal US, bladder US, urodynamic testing, and serum creatinine within the first 3 months of life, with initiation of clean intermittent catheterizations (CIC) and addition of antimuscarinic therapy when results suggest its role. These evaluations are repeated throughout childhood and adulthood [37]. When required, there are surgical interventions which may be offered as well. Cutaneous vesicostomy, suprapubic cystostomy, cystostomy button placement, external sphincterotomy, injecting botulinum toxin to the sphincter, ileovesicostomy, and incontinent intestinal diversions may be used in the setting of incontinence [38]. Botulinum toxin injections to the detrusor, bladder augmentation, and placement of a continent catheterizable channel (Mitrofanoff) may also be used in the setting of incontinence [34]. Whether conservative or surgical, the goals for neurogenic bladder management include protection of renal function and achievement of social independence to improve quality of life [34,37,38].

#### 1.7.3. Neurogenic Bowel

Many individuals with SB experience neurogenic bowel as well, which may be associated with constipation or incontinence [39,40]. Neurogenic bowel occurs due to alterations in anatomy, abnormal sensation, impaired colonic transit, and compromised innervation similar to that of neurogenic bladder [41]. This may be further confounded by cognitive deficits as well. Lesions above the conus medullaris lead to UMN (hyperreflexia) dysfunction, resulting in the failure of evacuation and impaction, while lesions at or below this level leads to LMN (flaccid) dysfunction resulting in incontinence. As such, the management focuses on the expected bowel patterns. Interventions range from nutritional and lifestyle management to conservative medications, or, as a last resort, surgical interventions. Increasing fiber and fluid intake is effective for improving stool consistency. Laxatives may improve consistency and increase colonic transit. Suppositories are quickly absorbed and can correct irregular emptying via chemical reflexive evacuation of the rectal vault and distal colon. If individuals require a more aggressive approach, transanal irrigation or continence catheter may be used. Other surgical options include sacral nerve stimulation, segmental resection, or Malone anterograde continence enema (MACE) and diversion colostomy [41]. As with neurogenic bladder management, the goals for bowel management are daily bowel movements, protection of gastrointestinal system, as well as social continence and independence to improve quality of life. 

#### 1.7.4. Nutrition

Individuals with SB usually have high rates of overweight and obesity during both childhood and adulthood [42]. This may be further exacerbated by poor eating habits, decreased physical activity, and lower resting energy expenditure associated with lower fat-free mass [42,43]. As a result, they are at increased risks for the metabolic syndrome, including diabetes mellitus, dyslipidemia, hypertension, cardiovascular disease, sleep apnea, osteoporosis, and poor skin integrity [27,37]. Management includes early lifestyle modifications, such as decreasing total daily caloric intake, decreasing intake of processed food, avoiding sugar and late night snacks, 8–10 h of sleep per night, and regular exercise [37,42]. Children over the age of 6 should engage in 60 min or more of physical activity daily with vigorous aerobic activity at least 3 days per week [27,44].

#### 1.7.5. Orthopedics

Individuals with SB commonly encounter orthopedic issues due to muscle imbalances and weakness, resulting in abnormal growth and deformities. These abnormalities are frequently seen in the spine and lower extremities and tend to be associated with the level of neurologic impairment. Orthopedic intervention should be adapted to the needs and goals for each individual patient.

Neuromuscular scoliosis is reported to be present in up to 52% of patients with SB and is more common in non-ambulatory individuals with higher lesions [45]. The presence of kyphosis and scoliosis may impact sitting balance, cause abnormal weight distribution increasing risk of pressure ulcers, compromise respiratory capacity, cause pain or negative body image concerns, and limit function. Once detected, close surveillance is introduced to monitor progression and need for intervention. Evidence of rapid progression should trigger concerns for tethered cord or syrinx, and neurosurgical consultation should be sought. Bracing has not been shown to be effective in the management of neuromuscular scoliosis, but it often recommended prior to surgery [46]. Surgical options for neuromuscular scoliosis include spinal fusion or growing rods, although there is only limited evidence of long-term benefits in the SB population; the high risk to benefit ration needs to be carefully considered [45,46,47]. Additionally, those with comorbid pulmonary function compromise are at increased risk of post-operative respiratory complications [47].

Neuromuscular hip subluxations and dislocations are also commonly seen in SB and require close monitoring; they are typically associated with weak or absent hip extensors and abductors, but active hip flexors and adductors. While it was previously thought that hip placement correlated with function, gait studies have since shown that it the presence of hip contractures more likely affect ambulation than hip placement [48]. As such, surgical intervention is not recommended at this time, with exception of unilateral dislocations and low lumbar or sacral lesions [37,48]. Unilateral dislocations tend to cause pelvic obliquity, which may lead to scoliosis progression and difficulty with seating systems. 

Additional lower extremity orthopedic abnormalities include contractures and rotational deformities of the femurs, tibias, knees, and feet associated with muscle imbalances. Nonsurgical approaches, including stretching, bracing, de-rotational straps, serial casting, and soft tissue releases are considered before proceeding with the more extensive osteotomies and fusions for these deformities. Surgical interventions are only recommended if the abnormalities are limiting or interfering with function [49]. Foot abnormalities usually include equinovarus and calcaneus deformities. Ponseti casting and tendon releases are recommended for clubfoot or congenital talus deformities with the goal of promoting plantigrade feet for weightbearing or bracing to protect soft tissues [50].

Osteoporosis is frequently seen below the level of the lesion and fractures are commonly seen due to smaller bones, lower bone mass, and mineralization deficits associated with endocrinologic and metabolic abnormalities, particularly during critical periods of bone deposition [51,52]. Those with higher SB lesions who are non-ambulatory tend to have decreased activity levels, higher body fat levels, and more contractures, all lending to an increased fracture risk [52]. Fractures are more commonly reported in children than adults and typically involve the distal femur and tibia or femoral neck [53]. It is recommended that children receive adequate calcium and vitamin D and participate in daily weight-bearing activities to promote bone health; some also undergo bisphosphonate infusions [37].

#### 1.7.6. Rehabilitation

Pediatric rehabilitation physicians are crucial throughout the lifespan of individuals with SB, starting with prenatal counseling and continuing through the transition to adulthood. The largest roles and goals of the rehabilitation physician is to facilitate developmental milestones and assist with the development of treatment plans that foster independence and success for the individuals and families in all areas of life [26]. Challenges may become more apparent as a child grows and develops, and these may be medical, physical, cognitive, or behavioral. Initially, rehabilitation physicians assist with habilitation, optimizing function with current conditions by working closely with physical and occupational therapists, speech-language pathologists, orthotists, and durable medical equipment vendors [26]. There is ongoing assessment of milestone attainment and interventional services are provided as indicated. Additionally, the pediatric physiatrist closely monitors function, assessing the neurologic level at each visit. As such, they are often the first to catch changes in muscular strength, sensation, or emergence of abnormal tone, warranting immediate diagnostic workup. Many individuals with SB experience medical or surgical setbacks that then require further rehabilitation [54].

#### 1.7.7. Neuropsychological

The outcomes of SB extend beyond physical symptoms. Children with SB often exhibit neurocognitive deficits that affect their abilities at school and may manifest as emotional and psychological distress [54]. As previously mentioned, higher SB lesions are associated with more severe brain malformations, higher rates of intellectual disabilities and poorer outcomes [26,54]. Decreased health-related quality of life, discrimination from peers, decreased school attendance, and lower educational attainment and employment have been reported among those with SB. They may also demonstrate reduced social interactions, contributing to difficulty in social development and increase in anxiety and depressive symptoms [55]. Of note, in addition to annual medical evaluations, these children and their families should be provided an individualized educational plan (IEP) as they progress through the educational system, and an individualized transition plan as they approach adulthood to provide increasing independence, autonomy, and personal responsibility for health-related tasks [56]. It is important to routinely address these psychosocial concerns and provide the appropriate neuropsychological and psychological support for this patient population throughout their lifetime [24,26,27].

## 2. Pediatric Spinal Cord Injuries

### 2.1. Introduction & Epidemiology

Compared to adults, spinal cord injuries (SCI) are relatively rare in the pediatric population. They can occur due to traumatic or nontraumatic causes. The incidence of pediatric traumatic SCI has been estimated to be between 17.5–26.9 cases per million in the United States [57,58], and appears to occur more frequently in adolescents than children [59]. As with adults, males are more likely to have a traumatic SCI than females; however, there is less disparity in the pediatric population [57]. Cervical SCI is the most common level for children and adolescents, representing approximately 40% of all injuries, followed by thoracic (~21%), lumbosacral injuries (16%), and 22% that are unspecified; the majority of pediatric traumatic SCIs result in incomplete injuries [57].

### 2.2. Pathophysiology & Epidemiology

Children demonstrate anatomical and physiological differences compared to adults, with vertebral columns have incomplete ossification with larger disc spaces, unossified vertebrae, shallow angle facets, and lax ligaments. These characteristics allow for the vertebral column to be more tolerant to stress than the spinal cord, which contributes to spinal cord injuries in the pediatric population. Infants and toddlers have a large head to body ratio, leading to a greater fulcrum force and resulting in cervical injuries more easily than adults [60]. When injuries occur to the lumbar spine, it is often due to lap belt injuries. To prevent this, there are car seat and booster seat regulations. The purpose of booster seats is to help keep the lap belt below the pelvic rim and the shoulder belt off the neck and over the clavicle laterally [61].

Most pediatric SCIs are caused by motor vehicle collisions (MVCs), followed by falls, being struck by objects, firearm injuries, sports, and other etiologies. More focused analysis demonstrates pediatric SCIs were most commonly due to MVCs in children younger than 5 years, due to falls in ages 6–12 years, and being struck by objects in ages 13–15 years. Sports injuries were most frequent in the 6–15 age range [57]. Recent data from the 2016 Kids’ Inpatient Database demonstrated that older, male, and black children are disproportionately burdened by traumatic SCI, and black children were more likely to have a diagnosis of traumatic SCI resulting from a firearm incident or assault compared to white children [62].

Similar to SB, children and adolescents with spinal cord injuries demonstrate multisystem and comprehensive involvement. However, in combination with abnormalities in growth and development, pediatric spinal cord injuries exhibit additional complications not seen in adults [54]. The maturity of the spine and spine-supporting structures are important variables distinguishing spinal cord injuries in children compared to adults [63].

### 2.3. Classification

The International Standards for Neurological Classification of SCI (ISNCSCI) are used to classify neurologic levels of injury in both adults and children [64]. Before the examination is initiated, the child and caregiver should be well-informed of what to expect [65,66]. When using the ISNCSCI, examiners use modifications by making it fun and using age-appropriate terminology for the pediatric population. A doll demonstration may be used to help illustrate the exam. Often, sensory level is being approximated by hiding the child’s vision and assessing response to the touch of pin or cotton swab and strength is observed with voluntary movements; preselected dermatomes can provide an estimate of a full exam [67].

SCI syndromes are the same as those seen in adults, with the exception of spinal cord injury without radiographic abnormality (SCIWORA), a pediatric phenomenon. SCIWORA in children was first defined when children presented with signs of myelopathy without evidence of injury on X-ray or computed tomography (CT) imaging [68]. With the development of MRIs, however, the damage to the spinal cord and surrounding soft tissue structures are more easily seen [69]. SCIWORA is thought to be a unique entity in children due to the anatomy of the developing spine, including underdeveloped muscles, ligamentous laxity, shallow and horizontally oriented facet joints, anterior wedging of vertebral bodies, and the large head-to-body ratio [70]. As a result of these characteristics, the pediatric vertebral column is more flexible than the spinal cord, lending to injury [71].

### 2.4. Common Comorbidities

Complications seen in pediatric SCI are similar to those described above for children with SB, including tethered cords and syrinx as they grow, neurogenic bowel and bladder, neuromuscular deformities, and metabolic and endocrinologic impairments. Fortunately, they are not predisposed to hydrocephalus, Chiari II malformations, nor cognitive dysfunction. Nonetheless, they are at risk of the same complications frequently experienced in adult SCI, including neurogenic bradycardia, orthostatic hypotension (NOH), autonomic dysreflexia (AD), cardiometabolic dysfunction, neurogenic restrictive lung disease, neurogenic obstructive lung disease, neuropathic pain, spasticity, neurogenic bladder, neurogenic bowel, osteoporosis, and heterotopic ossification, all of which are discussed in detail in manuscripts included in this special issue. The important differences to mention are those in vital signs and bladder capacity that are age dependent. For infants, blood pressures are lower and heart rates are faster; as children age, these parameters stabilize with approaching adulthood [72]. Age-appropriate vitals have to be considered when monitoring for AD and NOH. The majority of children with AD present with facial flushing, headaches, sweating, and tachycardia; interventional strategies are similar to those for adults [72,73,74]. Bladder capacity increases with age as well so pediatric maximum bladder volumes are smaller. When children have SCI at a young age, they typically have a reduced bladder capacity compared to their non-SCI peers because bladders do not hypertrophy due to changes in bladder wall compliance and spasticity [75]. Additionally, children have a longer period of time at risk of developing renal damage if SCI occurs early in life.

## 3. Conclusions

Despite the lesions or injuries, individuals with SB or SCI are able to live full and productive lives with appropriate clinical care. They should be monitored closely with a multi-disciplinary team approach and provided the appropriate tools, opportunities, and resources to allow them to succeed at any age. Frequent surveillance is required early on and as the child grows, since traction on central nervous system elements, and the effects of gravity on paralyzed truncal and abdominal musculature, puts them at high risk of tethered cord, Chiari II dysfunction, neuromuscular scoliosis and worsening respiratory dysfunction. These potential comorbidities also increase the risk of worsening autonomic dysfunction, neurogenic bladder dysfunction, neurogenic bowel dysfunction, spasticity, pressure injuries and social isolation. Finally, individualized transition plans to adulthood are essential for increasing independence, autonomy, and community integration.

## Data Availability

Not applicable.
